# Ultrafast Charge Transfer 2D MoS_2_/Organic Heterojunction for Sensitive Photodetector

**DOI:** 10.1002/advs.202207743

**Published:** 2023-02-19

**Authors:** Zhuhua Xu, Miao He, Qinke Wu, Chengcheng Wu, Xubiao Li, Bilu Liu, Man‐Chung Tang, Jie Yao, Guodan Wei

**Affiliations:** ^1^ Tsinghua‐Berkeley Shenzhen Institute (TBSI) Tsinghua University Shenzhen 518055 China; ^2^ Institute of Materials Research Tsinghua Shenzhen International Graduate School (SIGS) Tsinghua University Shenzhen 518055 China; ^3^ Shenzhen Geim Graphene Center Tsinghua Shenzhen International Graduate School (SIGS) Tsinghua University Shenzhen 518055 China; ^4^ Department of Materials Science and Engineering University of California, Berkeley Berkeley CA 94720 USA

**Keywords:** charge transfer, monolayer MoS_2_, organic thin film, photodetector, P–N heterojunction

## Abstract

The 2D MoS_2_ with superior optoelectronic properties such as high charge mobility and broadband photoresponse has attracted broad research interests in photodetectors (PD). However, due to the atomic thin layer of 2D MoS_2_, its pure photodetectors usually suffer from inevitable drawbacks such as large dark current, and intrinsically slow response time. Herein, a new organic material BTP‐4F with high mobility is successfully stacked with 2D MoS_2_ film to form an integrated 2D MoS_2_/organic P–N heterojunction, facilitating efficient charge transfer as well as significantly suppressed dark current. As a result, the as‐obtained 2D MoS_2_/organic (PD) has exhibited excellent response and fast response time of 332/274 µs. The analysis validated photogenerated electron transition from this monolayer MoS_2_ to subsequent BTP‐4F film, whereas the transited electron is originated from the A^−^ exciton of 2D MoS_2_ by temperature‐dependent photoluminescent analysis. The ultrafast charge transfer time of ≈0.24 ps measured by time‐resolved transient absorption spectrum is beneficial for efficient electron–hole pair separation, greatly contributing to the obtained fast photoresponse time of 332/274 µs. This work can open a promising window to acquire low‐cost and high‐speed (PD).

## Graphical abstract

The novel organic material BTP‐4F is stacked with monolayer MoS_2_ to form a heterojunction, facilitating efficient charge transfer as well as significantly suppressed dark current for the photodetector, which exhibits excellent responsivity of 3.2 A W^−1^, detectivity of 1.6 × 10^9^ Jones, high external quantum efficiency of 756%, and fast response time of 332/274 µs.

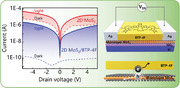

## 1 Introduction

The newly explored two‐dimension (2D) materials exhibit distinctive applications in advanced state‐of‐the‐art devices such as sensors,^[^ ^1^, ^2^, ^3^, ^4^, ^5^ ^]^ optical imaging,^[^ ^6^, ^7^, ^8^ ^]^ FET,^[^ ^9^, ^10^, ^11^, ^12^ ^]^ and photodetectors (PD)^[^ ^13^, ^14^, ^15^, ^16^, ^17^, ^18^, ^19^ ^]^ due to their excellent electrical and optical properties of high carrier mobility,^[^ ^20^, ^21^ ^]^ high absorption coefficient,^[^ ^22^ ^]^ suitable band gap,^[^ ^23^ ^]^ and strong light–matter interactions.^[^ ^24^ ^]^ The 2D transition‐metal dichalcogenides especially MoS_2_ with n‐type conductive feature due to nonmetallic defects, typically has a layer dependent band gap ranging from 1.2 to 1.9 eV, and could transfer to direct band gap when the number of layers reduced to monolayer,^[^ ^25^, ^26^ ^]^ indicating an ideal material choice for UV–visible and near infrared (NIR) photodetection. However, the conventional high‐vacuum chemical vapor deposition (CVD) or mechanically exfoliated 2D MoS_2_ film has inevitable drawbacks such as S vacancy,^[^ ^27^ ^]^ internal stress,^[^ ^28^ ^]^ and bound state,^[^ ^29^ ^]^ causing large dark current and weak photoresponse. The construction of 2D MoS_2_/inorganic heterojunction such as MoS_2_/WS_2_,^[^ ^30^ ^]^ MoS_2_/Graphene,^[^ ^31^ ^]^ and MoS_2_/BP^[^ ^32^ ^]^ is suitable to address these issues and these layered materials free of surface dangling bonds could provide a high‐quality interface or platform to promote the separation of photogenerated electrons and holes. Challenges remain to fabricate these all‐inorganic heterojunctions which require smoothly transferring the second 2D inorganic layers on the 2D MoS_2_ underneath without affecting their properties and continuity.

On the other hand, organic materials have significant merits of facile solution processability, lightweight conformation deposition, tailorable optoelectronic properties, and excellent flexibility. The vertical stacked P–N junction with P‐type high mobility organic materials and N‐type 2D MoS_2_ could construct functional multilayer structures through van der Waals (vdW) forces. The unique integration stack of inorganic/organic thin films could provide an energy favorable interface and structural design, facilitating effective charge transfer. Therefore, various choices of the organic materials have been explored to form suitable heterojunction such as monolayer MoS_2_/CuPc (ZnPc),^[^ ^33^, ^34^ ^]^ and monolayer MoS_2_/C_60_^[^ ^35^ ^]^ in respective fields for realizing low‐cost, sensitive and high‐speed PD. A P–N diode with monolayer MoS_2_ and organic BTBT‐SAM material has suppressed the dark current down to pA range.^[^ ^36^ ^]^ The vertically stacked 2D MoS_2_/CuPc based PD has realized profound responsivity of 3.0 × 10^3^ A W^−1^ and fast response time of 436 µs due to an ideal type II heterojunction interface created for ultrafast charge transfer.^[^ ^33^ ^]^ The monolayer MoS_2_/ZnPc diodes have demonstrated a response time of 8 ms and a high responsivity of 430 A W^−1^. The formed MoS_2_/ZnPc vdW interface is favorable to separate photogenerated holes to the ZnPc molecules, far away from the traps in MoS_2_ and the dielectric interface.^[^ ^34^ ^]^ Therefore, 2D MoS_2_/organic heterojunction is an appropriate candidate combination to realize (PD) with high sensitivities. Since all these organic films are thermally evaporated, efforts remain to explore facile solution processable organic materials for a new conductive hole transport layer (P‐type) to stack with 2D MoS_2_ layer, as well as maintaining an idealized interface for fast charge transfer.

P‐type solution‐processed narrow‐bandgap non‐fullerene acceptor materials of named as BTP‐4F ((2,20‐((2Z,20Z)‐((12,13‐bis(2ethylhexyl)‐3,9‐diundecyl‐12,13‐dihydro‐[1,2,5]thiadiazolo[3,4‐e]thieno[2′,30′:4′,50]thieno[20,30:4,5]pyrrolo[3,2g]thieno[20,30:4,5]thieno[3,2‐b]indole‐2,10‐diyl)bis(methanylylidene))bis(5,6‐difluoro‐3‐oxo‐2,3‐dihydro‐1H‐indene‐2,1‐diylidene))dimalononitrile), have been extensively studied as a non‐fullerene acceptor for organic photovoltaic research, boosting single junction organic solar cell efficiency around 19%.^[^ ^37^ ^]^ Therefore, the BTP‐4F with narrow band absorption at near‐infrared (NIR) region is a novel organic small molecule for optoelectrical devices. Due to the sterically hindering of central conjugated core, the over‐aggregation of the nitrogen atoms which located at the alkyl side chains can be limited, forming intramolecular charge transport channel. On the other hand, the BTP‐4F can be dissolved in common organic solvent such as chloroform and tetrahydrofuran at room temperature and its thermal decomposition temperature is as high as 318 °C, becoming an ideal candidate for solution‐processable organic material. Meanwhile, the BTP‐4F has very low exciton energy which is beneficial for efficient exciton separation.^[^ ^38^ ^]^ In this work, BTP‐4F thin film has been successfully integrated with monolayer 2D MoS_2_ to construct a suitable PN junction. Owing to the heterojunction formed at the interface of the inorganic/organic films, the dark current of as‐obtained (PD) is substantially suppressed by more than four orders of magnitude while the photoresponse time upon light excitation is significantly reduced from 1.2 s to 332 µs. The heterojunction (PD) exhibits excellent responsivity of 3.2 A W^−1^, detectivity of 1.6 × 10^9^ Jones, high external quantum efficiency (EQE) of 756% at a bias voltage of ≈5 V. The significant photoluminescence (PL) quenching signals indicate obvious charge transfer at the interface of the formed 2D MoS_2_/BTP‐4F heterojunction. The Kelvin probe force microscope (KPFM) demonstrates the effective electron charge transfer from monolayer MoS_2_ to BTP‐4F film. The temperature‐dependence PL indicates the transited electrons are from the A^−^ exciton by observing of changes in A exciton binding energy. The transient absorption (TA) spectra indicate the ultrafast charge transfer process occur in the interface. This innovative work has provided an organic‐modulated 2D (PD) for next generation detection with high sensitivities and fast response speed.

## 2 Results and Discussion

The CVD grown monolayer MoS_2_ is prepared on Si/SiO_2_ substrate as the triangular single crystal. The small organic molecule BTP‐4F of Figure S1a (Supporting Information) is chosen to build monolayer MoS_2_/BTP‐4F heterojunction by spin‐coating method. The monolayer MoS_2_ is consisted of two S and one Mo atoms and its thickness is characterized by atomic force microscope (AFM) as shown in Figure S1b (Supporting Information), indicating the thickness is 0.8 nm which is consistent with the reported result of high‐quality monolayer MoS_2_.^[^ ^39^ ^]^ Figure S1c (Supporting Information) shows the AFM image of BTP‐4F film by spin‐coating method on the Si/SiO_2_ substrate and the *insert* is the height curve taken from the region marked with dotted line which stretches across the groove marked artificially, and exhibits the thickness of BTP‐4F film is 32 nm. The monolayer MoS_2_ can emit stronger PL compared with the multilayer MoS_2_, because as number of layers decreasing to monolayer, 2D MoS_2_ will transfer from indirect band gap to direct band gap. Figure S1d,e shows (Supporting Information) the optical image and corresponding PL mapping of monolayer MoS_2_, and the single crystal with triangle marked with white arrow in Figure S1e (Supporting Information) shows the uniform PL intensity, in contrast, the PL intensity of irregular polycrystalline monolayer MoS_2_ marked with yellow arrow is unevenly distributed. Figure S1f (Supporting Information) shows the SEM of the single crystal of monolayer MoS_2_ with triangle after spin‐coating BTP‐4F film.

**Figure 1:**
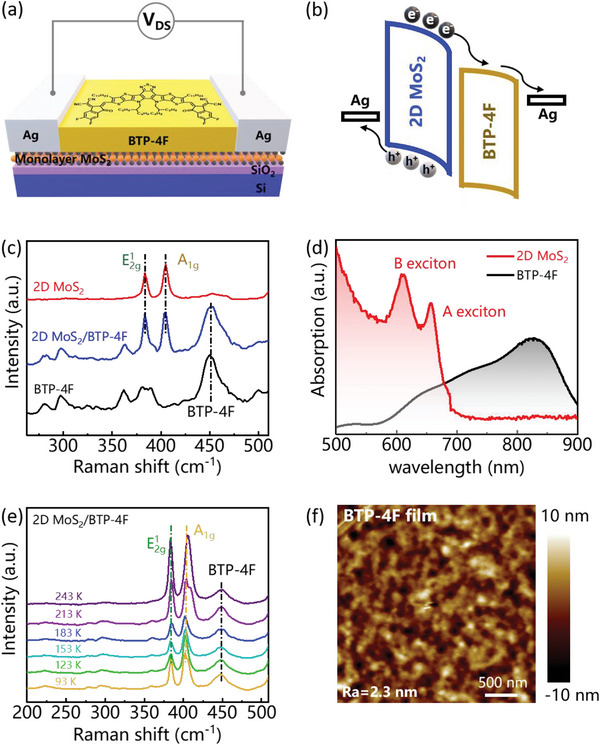
a) The illustrate of monolayer MoS_2_/BTP‐4F device. b) The energy level marching for the monolayer MoS_2_ and BTP‐4F film at the *V*_DS_ of 5 V. c) The Raman spectra corresponding to neat monolayer MoS_2_ and BTP‐4F, and 2D MoS_2_/BTP‐4F heterojunction. d) The absorption spectra corresponding to neat monolayer MoS_2_ and BTP‐4F, respectively. e) The temperature‐dependent Raman spectra of 2D MoS_2_/BTP‐4F heterojunction. f) AFM image of BTP‐4F film.

The Ag electrodes are deposited by high‐vacuum thermal evaporation on the triangle monolayer MoS_2_ to obtain the transistor‐type PD, followed by spin‐coated BTP‐4F film to construct 2D MoS_2_/organic heterojunction. **Figure** **1**a shows the illustration of monolayer MoS_2_/BTP‐4F heterojunction (PD). The energy level alignment is shown in Figure 1b, indicating photogenerated electron transfer path from BTP‐4F, and holes from 2D MoS_2_ to Ag electrode. To note, the shifted energy levels between MoS_2_ and Ag electrode upon applied external voltage of *V*_DS_ facilitate the electron and hole charge collection. For comparison, the intrinsic energy level without any applied voltage was shown in Figure S1g (Supporting Information). Figure 1c shows the Raman spectra corresponding to neat monolayer MoS_2_, BTP‐4F, and monolayer MoS_2_/BTP‐4F heterojunction. The transverse vibration mode E_2g_^1^ in the plane and longitudinal vibration mode A_1g_ out of plane are sensitive for the number of layers. The Raman shift peaks of E_2g_^1^ and A_1g_ for the neat monolayer MoS_2_ are located at the 383.5 and 404.1 cm^−1^, respectively,^[^ ^40^ ^]^ which is the typical Raman shift positions of monolayer MoS_2_, indicating the 2D MoS_2_ sample we used to fabricate device is the monolayer. The Raman shift of monolayer MoS_2_/BTP‐4F heterojunction exhibits the peaks of E_2g_^1^ and A_1g_, which are the same position before the BTP‐4F spin‐coating, demonstrating the structure of monolayer MoS_2_ is not broken by the process of introducing of BTP‐4F. Also, the Raman shift peaks of BTP‐4F occurs at the 2D MoS_2_/BTP‐4F heterojunction, indicating the successful BTP‐4F deposition on the surface of monolayer MoS_2_. Figure 1d plots the absorption spectra of neat monolayer MoS_2_ and BTP‐4F. The two absorption peaks of 657 nm and 611 nm correspond to the A and B excitons of monolayer MoS_2_, and the BTP‐4F absorption mainly locates at the range of 700–900 nm. The temperature‐dependent Raman spectra of Figure 1e ranging from 93 to 243 K shows that the three critical peaks barely shift, indicating stable feature of as‐obtained 2D MoS_2_/BTP‐4F thin film. Figure 1f shows continuous BTP‐4F film with roughness as small as 2.3 nm, indicating continuous and smooth surface coverage.

**Figure 2:**
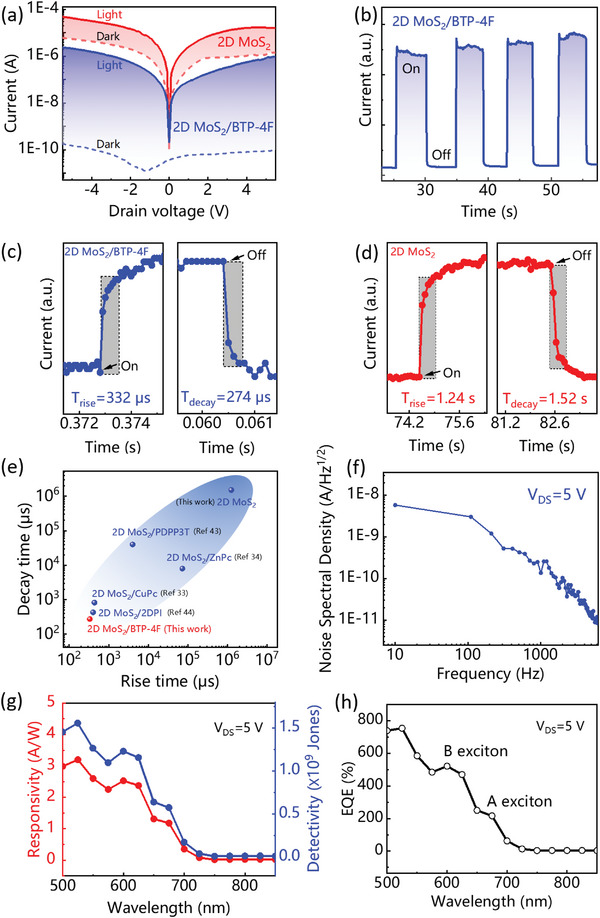
a) The *I–V* curves corresponding to pure MoS_2_ and MoS_2_/BTP‐4F devices, respectively. b) The on/off measurement of MoS_2_/BTP‐4F devices. c) The rise/decay time of the MoS_2_/BTP‐4F devices. d) The rise/decay time of the pure MoS_2_ device. e) The rise/decay time comparison based on published results in literatures and this work. f) The noise spectral density versus measured frequency. g) The responsivity, detectivity and h) EQE of MoS_2_/BTP‐4F device as function of wavelength, respectively.

To test the device photoelectric property, the current–voltage (*I–V*) curves are measured with the drain voltage sweeping from −5 to 5 V (*V*_DS_) under the 630 nm incident illumination as shown **Figure** **2**a. The *I–V* curves exhibit closely linear and symmetrical feature, indicating ohmic contact was formed between monolayer MoS_2_ and Ag electrodes. The dark current of 2D MoS_2_/BTP‐4F heterojunction device is more than four orders of magnitude lower than that of neat monolayer MoS_2_ device, indicating effective built‐in potential barrier. The photocurrent of heterojunction is an order of magnitude lower after introducing of BTP‐4F film. Compared with neat monolayer MoS_2_ devices, the on/off ratio of monolayer MoS_2_/BTP‐4F heterojunction device is increased by ≈1000 times as shown in the Figure S2 (Supporting Information), indicating strong coupling effect of BTP‐4F and monolayer MoS_2_. Due to the intrinsic defects and big surface‐to‐volume ratio which result in surface bound state, the response speed of neat monolayer MoS_2_ phototransistor still stay in the level of several and even tens of seconds^[^ ^41^, ^42^ ^]^ which limits the application of 2D materials in (PD) as shown in Figure 2d. Surprisingly, the response speed of monolayer 2D MoS_2_/BTP‐4F heterojunction device as shown in Figure 2c reaches the level of microsecond compared with that of second for neat monolayer MoS_2_ device. The response time for the photodetector is including the rise and fall time, defined as the time taken between 10% and 90% of the maximum value of photocurrent.^[^ ^43^ ^]^ Figure S3 (Supporting Information) and 2b shows the *current–time* (*I–t*) curves corresponding to the PD of neat monolayer MoS_2_ and 2D MoS_2_/BTP‐4F heterojunction, respectively. The two devices exhibit the *current–time (I–t)* curves with square wave shape under the light control, indicating the light response. Obviously, the current–time (*I–t*) exhibits tidier square wave shape after introducing of BTP‐4F film, indicating 2D MoS_2_/BTP‐4F PD owns more sensitive photoelectric response. The *I–t* curves for the 2D MoS_2_/BTP‐4F PD after remaining idle in air for 6 and 12 hours are shown in Figure S4 (Supporting Information), indicating operation stability for as‐obtained PDs without any encapsulation. To note, the 2D MoS_2_/BTP‐4F PD has response time as fast as 332/274 µs (Figure 2c), which is about three orders of magnitude faster compared with pure 2D MoS_2_ PDs of 1.24/1.52 s (Figure 2d). As shown in Figure 2e, the as‐obtained 2D MoS_2_/BTP‐4F PD has the fastest response speed compared with similar device structure in the literatures.^[^ ^33^, ^34^, ^44^, ^45^ ^]^

The responsivity (*R*), detectivity (D*), and the external quantum efficiency (EQE) are key indicators of PD performance and calculated according to the formula

(1) $$R=\frac{I_{\mathrm{light}}-I_{\mathrm{dark}}}{PA}$$

(2) $$D^{*}=\frac{R\sqrt{A\Delta f}}{I_{N}}$$

(3) $$\mathrm{EQE}=\frac{Rhc}{e\lambda }$$

where *I*_light_ and *I*_dark_ are light and dark currents, respectively. *P*, *A*, and *e* represent the power density of excitation light, channel effective area of PD, and electronic charge, respectively. Δ*f* and *I*_N_ refer the electrical bandwidth of the noise measurement and noise current, respectively. *h*, *c*, and 𝜆 represent Planck constant, the speed of light and the light wavelength. Figure 2f shows the low noise spectral density gradually reduces as the test frequency is gradually increased to 6000 Hz, indicating low noise current. Figure 2g exhibits the responsivity and detectivity as function of wavelength from 500 to 850 nm under the uniform light power and the bias voltage of 5 V and the maximum can be reached 3.2 A W^−1^ and 1.6 × 10^9^ Jones. The high response mainly locates in the range of monolayer MoS_2_ absorption. In contrast, the absorption range of BTP‐4F doesn't make contribution to the response, indicating the response is mainly from the monolayer MoS_2_. Figure 2h plots the EQE dependence on wavelength and the maximum value is up to 756% under the drain voltage of 5 V, whereas the two peaks of 611 and 657 nm are consistent with the A and B excitons as‐obtained monolayer MoS_2_ as shown in Figure 1d. The applied bias has possibly increased photomultiplication effect of photogenerated carriers, resulting larger than 100% of EQE. The value of EQE more than 100% range covers the 500–700 nm of Figure 2h, indicating the as‐obtained (PD) is suitable for the broad visible light spectral response. Due to the horizontal device structure as shown in Figure 1a, the as‐coated organic layer of BTP‐4F is directly connected with Ag electrode and no extra electron transport layer has been incorporated to facilitate electron transport. Therefore, the electron charge collection efficiency has been hindered by the potential barrier between Ag and BTP‐4F. As a result, the photocurrent generation upon organic layer absorption become weaker compared with 2D MoS_2_ as shown in Figure 2h. Photocurrent linearly increase with the varied incident laser power as shown in Figure S5 (Supporting Information), indicating photogenerated electron–hole pairs have been effectively dissociated and separated without obvious nonradiative recombination.

**Figure 3:**
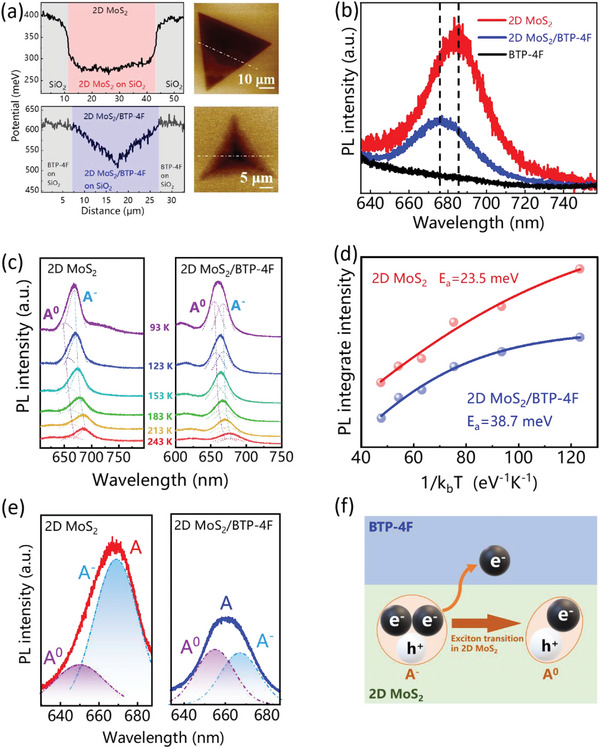
a) The potential–distance curves extracted from the region marked with white dotted lines in corresponding potential mapping tested by KPFM of monolayer MoS_2_ without and with BTP‐4F film, respectively. b) The PL spectra corresponding to neat monolayer MoS_2_ and 2D MoS_2_/BTP‐4F heterojunction, respectively. c) The temperature‐dependent PL spectra corresponding to neat monolayer MoS_2_ and 2D MoS_2_/BTP‐4F heterojunction, respectively. d) The PL integrate intensity as function of 1/*k*_b_ *T* corresponding to neat monolayer MoS_2_ and 2D MoS_2_/BTP‐4F heterojunction, respectively. e) The PL peaks of monolayer MoS_2_ and 2D MoS_2_/BTP‐4F fitted to A^0^ and A^−^ excitons at 93 K. f) The illustration of A^−^ transforming A^0^ exciton.

The 2D MoS_2_/BTP‐4F PD exhibits ultrafast response speed with the rise time of 332 µs, which is the fastest response speed for the 2D MoS_2_ based PDs to our best knowledge. Meanwhile, the dark current has been significantly reduced by four orders of magnitude to nA. To figure out the work mechanism and observe the details of charge transfer at the 2D MoS_2_/BTP‐4F heterojunction, the potential mapping of monolayer MoS_2_ without and with BTP‐4F film are tested by KPFM. The conversion process of potential and Fermi energy level as shown in the Supporting Information. **Figure** **3**a shows the potential–distance curve extracted from the region marked with white dotted line in corresponding KPFM mapping images. The lower potential means higher Fermi level for the monolayer MoS_2_ compared with the Si/SiO_2_ substrate. After BTP‐4F spin coating on the surface of monolayer MoS_2_, the potential–distance curve exhibits the potential of BTP‐4F film decreases for the overlapping region of monolayer MoS_2_ and BTP‐4F, therefore, the overlapping region at interfaces could own higher Fermi level due to possible free electrons diffused from monolayer MoS_2_ to BTP‐4F film. Meanwhile, the edge potential is higher than that of central region due to the influence of intrinsic defect states in monolayer MoS_2_. Figure 3b shows PL spectra corresponding to neat monolayer MoS_2_ and BTP‐4F, and 2D MoS_2_/BTP‐4F heterojunction. The PL peaks of neat monolayer MoS_2_ locate at ≈685 nm, which is corresponding to A exciton. There is no obviously PL emitting peak at the range of ≈600 to ≈750 nm for neat BTP‐4F. The intensity of PL for the 2D MoS_2_/BTP‐4F decreases obviously, indicating significant PL quenching which could be attributed to the subsequent formation of charge transfer channel in the interface of monolayer MoS_2_ and BTP‐4F film, and the appearance of blueshift was resulted from the change of exciton states.

The monolayer MoS_2_ is the main light absorption layer in which the A exciton consisted of biexciton (A^0^) and triexciton (A^−^).^[^ ^46^ ^]^ The biexciton, also called neutral exciton (A^0^) consists one hole in value band and one electron in conduction band, and the triexciton, also called negative exciton (A^−^) consists of one hole in valance band and two electrons in conduction band.^[^ ^47^ ^]^ The binding energy of triexciton is lower than that of biexciton, however there are still near, so they form the PL peak of A exciton together. And the binding energy corresponding mixed exciton can be calculated by the function^[^ ^34^ ^]^

(4) $$I_{T}=\frac{I_{0}}{1+A{\;e}^{\left(-\frac{E_{a}}{k_{b}T}\right)}}$$

where *I*_T_ represents the measured PL‐integrated intensities; e, *k*_b_, and *T* are natural constant, Boltzmann constant, and the measured temperature, respectively. *I*_0_ and *A* are the fitting parameters. The *E*_a_ refers to the binding energy of mixed excitons, which is determined by ratio of A^0^ and A^−^ excitons. If there are more A^0^ excitons available, the calculated binding energy will become higher. To calculate the corresponding binding energy of A exciton in the monolayer MoS_2_ with and without BTP‐4F film, the temperature‐dependent PL spectra are tested in Figure 3c. Meanwhile, there was PL peak blueshift after introducing of BTP‐4F under the test temperature ranging from 93 to 243 K. Figure 3d exhibits the PL integrated intensity as function of 1/*k*_b_ *T*, and the corresponding binding energy of mixed exciton is 23.5 meV for monolayer MoS_2_ and 38.7 meV for 2D MoS_2_/BTP‐4F heterojunction. The higher binding energy after introducing of BTP‐4F demonstrates the ratio of A^0^ exciton increases, which indicates that more and more free electron tends to be transferred to adjacent BTP‐4F film, and less A^−^ excitons remain. Figure 3e exhibits the A^−^ exciton located at ≈668 nm decreases in PL peak of A exciton after introducing of BTP‐4F, and the electrons transferring from monolayer MoS_2_ to BTF‐4F results in changing of A^−^ to A^0^ exciton located at ≈651 nm as shown in the illustrate of Figure 3f. And the peaks of A^−^ and A^0^ excitons exhibit the redshift as temperature increase as shown in Figure 3c.

**Figure 4:**
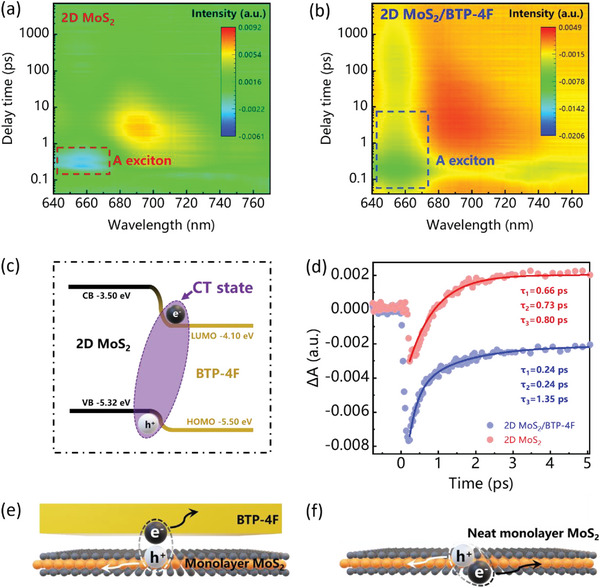
a,b) The TA spectra of neat monolayer MoS_2_ and 2D MoS_2_/BTP‐4F heterojunction. c) The illustrate of charge transfer in the interface of monolayer MoS_2_ and BTP‐4F film. d) The corresponding kinetic decay‐associated spectra (DAS) process at 658 nm. e,f) The processes illustrate of photogenerated carriers’ generation and migration for neat monolayer MoS_2_ and 2D MoS_2_/BTP‐4F heterojunction.

To observe the process of charge transfer in the interface of monolayer MoS_2_ and BTP‐4F film, the transient absorption (TA) spectroscopy as an effective tool has been carried out to study exciton generation, transfer, and recombination processes. The 400 nm is selected as the wavelength of pump (1 kHz, 100 fs, around 1 µJ cm^−2^ per pulse), and the probe range is 640 to 770 nm. The absorption peaks (negative signal) in TA spectra located at 658 nm called ground state bleaching corresponding to the absorption of A exciton in the monolayer MoS_2_. **Figure** **4**a,b shows the TA spectra corresponding to neat monolayer MoS_2_ and 2D MoS_2_/BTP‐4F heterojunction, and the A exciton is probed. Figure 4c shows the illustrate of charge transfer in the interface of monolayer MoS_2_ and BTP‐4F film. To further analyze the exciton state, the kinetic decay‐associated spectra (DAS) process at 658 nm for neat monolayer MoS_2_ and 2D MoS_2_/BTP‐4F heterojunction are fitted to show the three processes of exciton generation of 0.66 ps, transfer of 0.73 ps and recombination of 0.80 ps for neat monolayer MoS_2_, and exciton generation of 0.24 ps, transfer of 0.24 ps and recombination of 1.35 ps for 2D MoS_2_/BTP‐4F interface, respectively, as shown in Figure 4d. After introducing of BTP‐4F film, the transfer time becomes shorter and recombination time becomes longer, indicating successfully nonstructured energy offset for fast charge transfer. Therefore, the charge transfer of 0.24 ps for 2D MoS_2_/BTP‐4F indicates the ultrafast charge transfer process upon light incident,^[^ ^48^, ^49^, ^50^ ^]^ promoting effective electron–hole pair separation. According to the PL intensity quenching in Figure 3b, there is effective charge transfer between 2D MoS_2_ and BTP‐4F. While typical electron transfer occurs in the Van der Waals heterojunction^[^ ^51^ ^]^ could be as fast as hundreds of femtoseconds, the charge transfer time of 2D MoS_2_/BTP‐4F heterojunction reaches 0.24 ps. Therefore, without the organic layer of BTP‐4F, there is no effective interface for exciton dissociation and surface bind states could exist,^[^ ^34^ ^]^ and photogenerated electron–hole pairs could even have nonradiative recombine in neat 2D MoS_2_ PDs in Figure 4e, resulting very slow response time of 1.24 s. In contrast, the introduced organic layer of BTP‐4F (Figure 4f) could not only provide an effective interface for exciton dissociate, but also spatially separate electron and hole charges, followed by swiftly collected by their respective electrode, greatly contributing to the ultrafast response speed. In the meantime, the BTP‐4F can passivate the surface states of 2D MoS_2_, improving the response time. To note, though charge transfer time at the stacked interface films plays a critical role to determine the photoresponse time of the as‐obtained PD, other factors such as channel lengths, contact barriers, and charge mobilities could all impact response speed. Finally, the response time is short to 332/274 µs rise/decay time for the obtained 2D MoS_2_/BTP‐4F PD.

## 3 Conclusion 

In summary, the high‐performance 2D MoS_2_/BTP‐4F heterojunction PD is fabricated with ultrafast response time of 332/274 µs, high responsivity, detectivity and EQE of 3.2 A W^−1^, 1.6 × 10^9^ Jones, and 756% at *V*_DS_ = 5 V, respectively. The dark current is decreased by four orders of magnitude to level of pA, and the response time is shortened by three orders of magnitude to the level of microsecond after introducing of the BTP‐4F organic layer. The temperature‐dependent Raman spectrum analysis indicates the stable stacking of the 2D MoS_2_ and BTP‐4F layers in ambient condition without any encapsulation protection. The PL quenching analysis indicates that the favorable energy offset has been formed for effective charge transfer. The detailed Kelvin Probe Force Microscope (KPFM) has systematically validated photogenerated electron transition from this monolayer MoS_2_ to subsequent BTP‐4F film, whereas the transited electron was originated from the A^−^ exciton of 2D MoS_2_ by temperature‐dependent photoluminescent (PL) analysis. The ultrafast charge transfer time as fast as 0.24 ps occurs by time‐resolved TA spectrum, significantly contributing to the fast response time of 332/274 µs for the as‐obtained 2D MoS_2_/BTP‐4F heterojunction PD. Therefore, this work promotes the promising development of high‐performance 2D MoS_2_‐based inorganic/organic heterojunction for sensitive PD application.

## 4 Experiment Section

### Materials Characteristics

The photoluminescence (PL) and Raman spectra were taken on Microscopic confocal Raman spectrometer (Horiba Lab RAM HR800, America) at room temperature. The absorption spectra were tested from the HP 8453 spectrophotometer. The atomic force microscopy (AFM) and Kelvin probe force microscopy (KPFM) was taken by Dimension Icon (Bruker Innova, USA). The SEM images was taken from the field emission scanning electron microscope (JEOL‐7401). The transient absorption (TA) spectra were measured in Helios pump probe system (Ultrafast system LLC) combined with an amplified femtosecond laser system (Coherent), and under 400 nm excitation at 1 kHz, 100 fs, around 1 µJ cm^−2^ per pulse.

### Device Fabrication

The monolayer MoS_2_ was prepared by conventional chemical vapor deposition (CVD) method. First, sulfur powder (100 mg, 99.5%, Sigma‐Aldrich) and the mixed metal precursors of MoO_3_ (99.9%, Sigma‐Aldrich)/KI (99.9, Sigma‐Aldrich) were loaded into the growth chamber. Then, the temperature of the furnace was increased to 790 °C within 20 min. During the heating process, the sulfur powder was introduced into the growth chamber at 640 °C. When the temperature of the furnace reaches 790 °C, the growth process lasts 5 min. After the growth is finished, the furnace was naturally cooled down. The BTP‐4F was purchased from Vizuchem Co., Ltd (Shanghai, China). The Ag electrodes was deposited on monolayer MoS_2_ as 300 nm thick under 5 × 10^−4^ Pa with deposition rate of 2 Å s^−1^. Then, the BTP‐4F was dissolved in chloroform as 10 mg mL^−1^, and deposited through spin‐coting on the monolayer MoS_2_ with predeposited Ag electrodes. The device fabrication was finished after annealing treatment for 1 h at 80 °C.

### Device Test

All *I–V* and current–time curves measurements are carried out with a home‐build system at room temperature which consisted of the optical and electrical parts. The optical part includes SC‐pro and AOTF‐PRO produced by OYSL and can produce 430–1450 nm light which provide the illumination needed by photodetectors. The electrical part consists of probe and Keithley 2600B and can detect the photodetectors’ current. The noise current was tested by SR830 lock‐in amplifier (Stanford research systems) and SR570 low‐noise current preamplifier (Stanford research systems). The response time was extracted from oscilloscope. The frequency spectra were tested by oscilloscope with signal‐amplifier and the LED light source adjusted by signal generator.

## Conflict of Interest

The authors declare no conflict of interest.

## Supporting information

Supporting information

## Data Availability

Research data are not shared.
