# Supplementary material for: Ultrafast Charge Transfer 2D MoS2/Organic Heterojunction for Sensitive Photodetector
Source: Adv Sci (Weinh). 2023 Feb 19;10(12):2207743. doi: 10.1002/advs.202207743 (PMC10131850; doi:10.1002/advs.202207743)
Supplement: Supplementary file 1 — Supporting information [file ADVS-10-2207743-s001.pdf]

ADVANCED SCIENCE
Open Access

Supporting Information

for *Adv. Sci.*, DOI 10.1002/advs.202207743

Ultrafast Charge Transfer 2D MoS_2/Organic Heterojunction for Sensitive Photodetector

*Zhuhua Xu, Miao He, Qinke Wu, Chengcheng Wu, Xubiao Li, Bilu Liu, Man-Chung Tang, Jie Yao and Guodan Wei**

Supporting Information

Zhuhua Xu[1,2#], Miao He[1,2#], Qinke Wu[1,2], Chengcheng Wu[1,2], Xubiao Li[1,2], Bilu Liu[1,2,3], Man-Chung Tang[2], Jie Yao[4], Guodan Wei[1,2]*

[1]Tsinghua-Berkeley Shenzhen Institute (TBSI), Tsinghua University Shenzhen 518055, China
[2] Institute of Materials Research, Tsinghua Shenzhen International Graduate School (SIGS), Tsinghua University Shenzhen 518055, China
[3] Shenzhen Geim Graphene Center, Tsinghua Shenzhen International Graduate School (SIGS), Tsinghua University Shenzhen 518055, China
[4] Department of Materials Science and Engineering, University of California, Berkeley, CA

# These two authors have contributed equally to this work.
***Corresponding authors:** Guodan Wei: weiguodan@sz.tsinghua.edu.cn

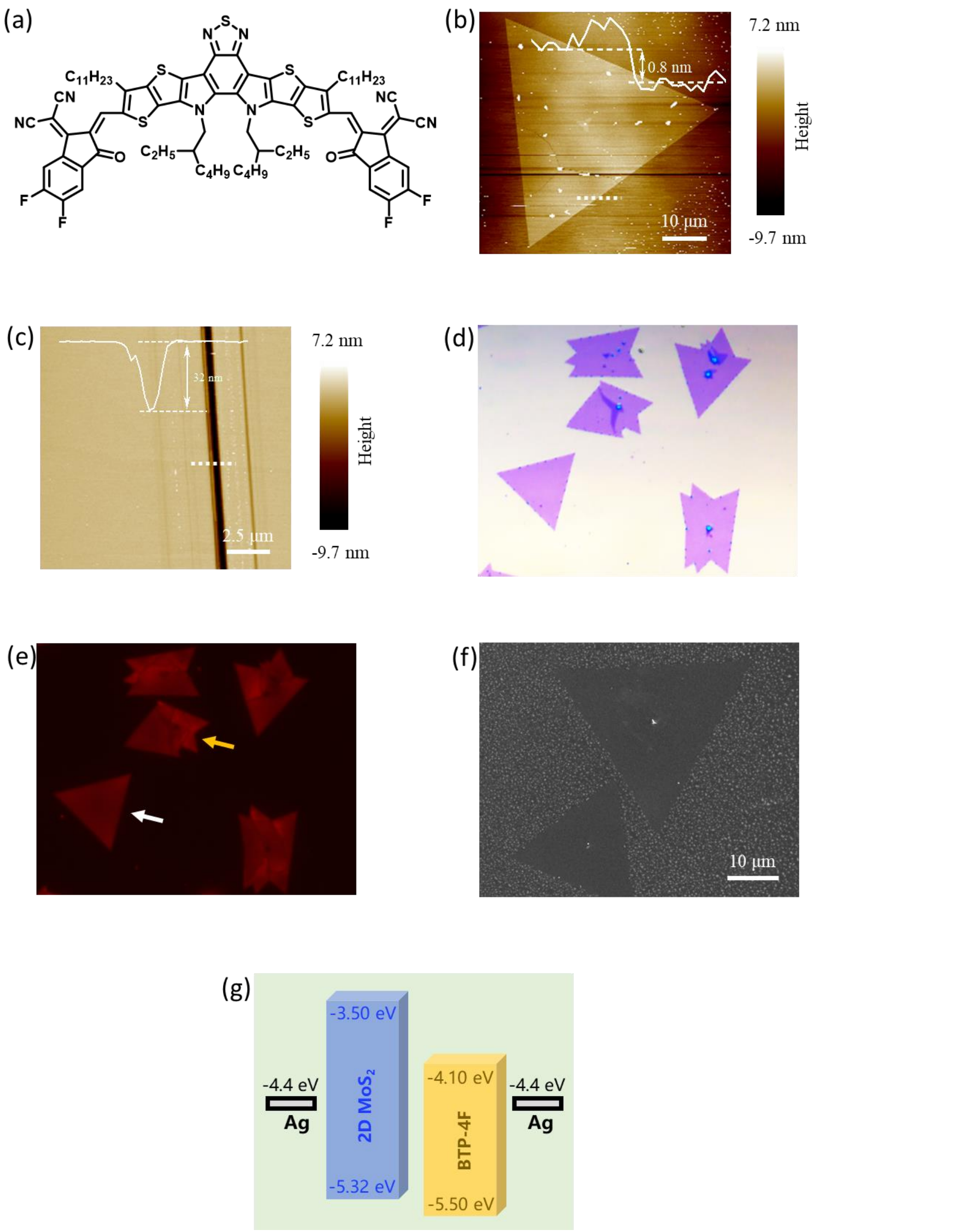

Figure S1 (a) The molecule of BTP-4F. (b) and (c) The AFM images of monolayer MoS_2 and BTP-4F film on the Si/SiO_2 substrate, respectively. Corresponding insert of (b) and (c): The height curve taken from the region marked with dotted line. (d) and (e) Optical image and corresponding PL mapping. (f) The SEM of monolayer MoS_2 after spin-coating BTP-4F. (g) The intrinsic energy level marching for the monolayer MoS_2 and BTP-4F film at the V_{DS} of 0 V

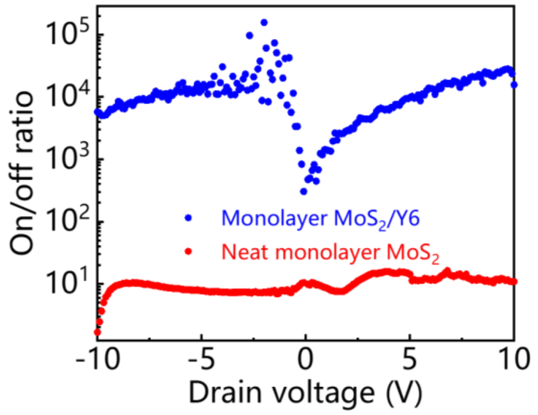

Figure S2 The on/off ratio of neat monolayer MoS_2 and 2D MoS_2/BTP-4F heterojunction photodetector, respectively.

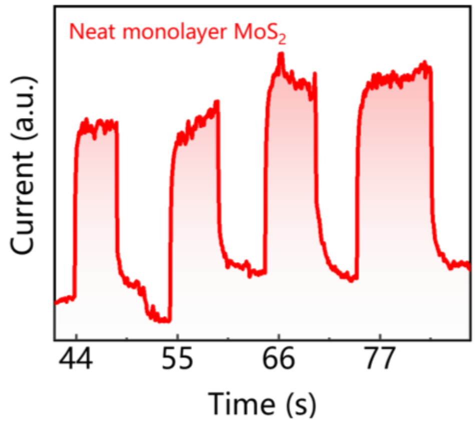

Figure S3 The time-current (*I*-*t*) curves of neat monolayer MoS_2 photodetector

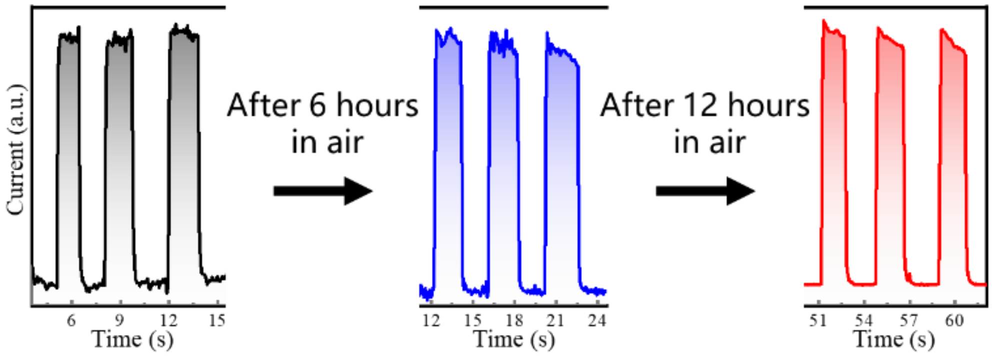

Figure S4 The time-current (*I*-t) curves of the 2D MoS_2/ BTP-4F photodetector after remaining idle for 6 and 12 hours in air

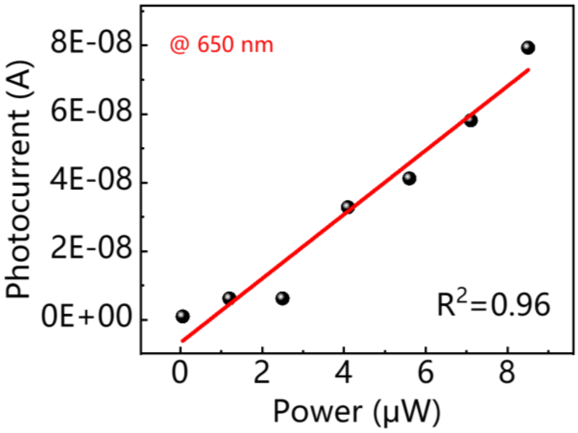

Figure S5 The photocurrent dependence on incident laser power on the 2D MoS_2/BTP-4F photodetector.

The Conversion process of potential and Fermi energy level

The potential difference between the AFM tip and sample surface can be calculated as follows:

$$eV_{tip-BTP-4F} = W_{tip} - W_{BTP-4F}$$

$$eV_{tip-MoS_2/BTP-4F} = W_{tip} - W_{MoS_2/BTP-4F}$$

where e, $W_{MoS2/BTP-4F}$, W_{BTP-4F} and W_{tip} represent electron charge, work functions of 2D MoS_2/BTP-4F heterojunction region, neat BTP-4F region, and AFM tip, respectively. So, the Fermi level difference (ΔE_f) between heterojunction and neat BTP-4F regions can be calculated by:

$$\Delta E_f = W_{BTP-4F} - W_{MoS_2/BTP-4F} = eV_{tip-BTP-4F} - eV_{tip-MoS_2/BTP-4F}$$
